# Experiences of a High-Risk Population with Prenatal Hemoglobinopathy Carrier Screening in a Primary Care Setting: a Qualitative Study

**DOI:** 10.1007/s10897-017-0159-7

**Published:** 2017-10-05

**Authors:** Kim C. A. Holtkamp, Phillis Lakeman, Hind Hader, Suze M. J. P. Jans, Maria Hoenderdos, Henna A. M. Playfair, Martina C. Cornel, Marjolein Peters, Lidewij Henneman

**Affiliations:** 1 Department of Clinical Genetics, Section Community Genetics, Amsterdam Public Health research institute, VU University Medical Center, PO Box 7057, 1007 MB Amsterdam, The Netherlands; 2 Department of Clinical Genetics, Academic Medical Center, Amsterdam, The Netherlands; 3 TNO, Quality of Life, Department of Child Health, Leiden, The Netherlands; 4 Midwifery practice Vida, Amsterdam, The Netherlands; 5 Midwifery practice Bijlmermeer, Amsterdam, The Netherlands; 6 Department of Pediatric Hematology, Emma’s Children’s Hospital, Academic Medical Center, University of Amsterdam, Amsterdam, The Netherlands

**Keywords:** Hemoglobinopathies, Sickle cell disease, Thalassemia, Carrier screening, Midwives, Primary care, Prenatal testing, Qualitative research

## Abstract

Carrier screening for hemoglobinopathies (HbPs; sickle cell disease and thalassemia) aims to facilitate autonomous reproductive decision-making. In the absence of a Dutch national HbP carrier screening program, some primary care midwives offer screening on an ad hoc basis. This qualitative descriptive study explores how pregnant women perceive an offer of HbP carrier screening by their midwife. Semi-structured interviews (*n* = 26) were conducted with pregnant women at risk of being a HbP carrier, and whom were offered screening at their booking appointment in one of two midwifery practices in Amsterdam. The results showed that half of the respondents were familiar with HbPs. Generally, women perceived the offer of HbP carrier screening as positive, and most women (*n* = 19) accepted screening. Seven declined, of whom two already knew their carrier status. Important reasons to accept screening were to obtain knowledge about their own carrier status and health of their unborn child, and the ease of the procedure. A multistep process of decision-making was observed, as many women did not give follow-up testing (e.g. partner, invasive diagnostics) much consideration while deciding on accepting or declining HbP screening. Women experienced information overload, and preferred receiving the information at a different moment (e.g. before the intake by a leaflet, or preconceptionally). In conclusion, while prenatal HbP carrier screening is perceived as positive, informed decision-making seems to be suboptimal, and both the content and timing of the information provided needs improvement.

**Electronic supplementary material:**

The online version of this article (10.1007/s10897-017-0159-7) contains supplementary material, which is available to authorized users.

## Introduction

Hemoglobinopathies (HbPs) such as sickle cell disease (SCD) and thalassemia are the most common autosomal recessive disorders worldwide (Williams and Weatherall 2012). HbPs are hereditary blood disorders characterized by severe anemia, and variable, but often high, morbidity and a shortened lifespan (Williams and Weatherall 2012). Though HbPs mostly occur in Africa, the Caribbean, Asia, the Middle East, and the Mediterranean area, global population movements have caused HbPs to be increasingly common in other parts of the world as well (Aguilar Martinez et al. 2014; Cao et al. 2002).

Genetic carrier screening for HbPs (either premarital, preconception or prenatal) allows couples to find out whether they are both HbP carriers and thus face a 1-in-4 risk of having an affected child each pregnancy. The primary aim of carrier screening is to enable carrier couples to make informed reproductive decisions, and is preferably done before pregnancy, as this maximizes reproductive options (de Wert et al. 2012; Henneman et al. 2016). Available options include: preimplantation genetic diagnosis, prenatal diagnosis, use of donor gametes, refraining from pregnancy, adoption or accepting the risk of having an affected child. Testing during pregnancy reduces the number of reproductive options, but may also inform the health professional about a coexisting anemia due to the HbP carrier status (Delatycki 2008; Jans et al. 2010). Moreover, since the treatment of coexisting anemia in HbP carriers needs a different approach from anemia in non-carriers during pregnancy, this is important information for the midwife. Iron suppletion is not the first treatment of choice when Hb is low in carriers since anemia may not be caused by an iron deficiency (Jans et al. 2010). Folic acid supplements should be given in the latter situation, unless a coexisting iron deficiency has been proven.

Worldwide, HbP carrier screening has been available for many years, and is carried out in a number of countries in different settings. As described by Locock and Kai 2008), ad hoc screening is the most common model used internationally, but more systematic carrier screening occurs for example in the United Kingdom (UK), Southern European and Middle Eastern Countries (Cao et al. 2002; Cousens et al. 2010; Locock and Kai 2008). In addition to the identification of children with a HbP, carrier status can be reported from neonatal screening as well. In that case parents may be referred to genetic centers for counseling, especially carrier couples, though it has been shown that few couples in the Netherlands are seen in genetic centers (Kaufmann et al. 2014). Screening offers also differ in the way of delivery (universal or ancestry-based), the timing (premarital, preconceptional or prenatal), its mandatory or voluntary character, and counseling and informed consent procedures (Cousens et al. 2014; Cousens et al. 2010; Giordano et al. 2014). Regarding this latter aspect, studies have shown that many people are unaware that they are being screened, as the test is usually presented as a routine blood test, and are often surprised or even shocked by test results (Cousens et al. 2013; Locock and Kai 2008).

The first HbP screening programs were aimed at thalassemia, and were developed during the 1970s in Mediterranean countries (Cao et al. 2002). In the Netherlands, as well as in many other countries, positive attitudes among individuals at risk for being a HbP carrier (Ahmed et al. 2002; Lakeman et al. 2009; Ross et al. 2011) and professionals (Jans et al. 2012; Tsianakas et al. 2010; Weinreich et al. 2009) towards premarital, preconceptional, and prenatal HbP carrier screening have been shown. It has been acknowledged that reaching at-risk couples before a pregnancy can be quite challenging, resulting in a limited success for *preconception* HbP carrier screening (Gustafson et al. 2007; Housten et al. 2016). An prenatal HbP carrier screening program linked to the newborn screening was therefore launched in the UK in 2004. In the UK, all women, regardless of ancestry, are offered carrier screening for thalassemia in early pregnancy. Additionally, in high prevalence areas, all pregnant women are also offered carrier screening for SCD and other hemoglobin variants, whereas in low prevalence areas, a Family Origin Questionnaire is first completed to assess the risk of women (National Health Services England 2012).

In 2006, the governing bodies of the World Health Organization adopted two resolutions on hemoglobin disorders, recommending, among other things, HbP screening and counseling programs (World Health Organization 2006a, 2006b). In the Netherlands, there is no national preconceptional and/or prenatal carrier screening program for HbPs: only newborn screening exists. In their advisory report in 2007, the Health Council of the Netherlands recommended a large feasibility and effectiveness pilot study of cystic fibrosis (CF) and HbP preconception carrier screening in conjunction with other aspects of preconception care (Health Council of the Netherlands 2007). Although this extended pilot has never been realized, some midwifery practices located in areas with relatively large populations at risk of being a HbP carrier, recognize the need for carrier screening, and thus offer screening on an ad hoc basis (i.e. on their own initiative in the absence of national policy support). However, due to the absence of national policy on HbP carrier screening, midwives do not operate in an uniform manner. Furthermore, studies in the Netherlands have mainly focused on attitudes towards HbP carrier screening (van der Pal et al. 2013; van Elderen et al. 2010), and very few have studied actual practice (Giordano et al. 2006; Kaufmann et al. 2011). To provide recommendations on the broadening and scaling up of carrier screening, these ad hoc initiatives should be studied. Furthermore, little is known about the experiences of high-risk women (i.e. women considered at higher risk of being a HbP carrier based on their ancestry) with HbP carrier screening in primary care. This study therefore explores how pregnant women perceive an offer of carrier screening for HbPs by their primary care midwife.

## Methods

A qualitative study design using semi-structured interviews was chosen, enabling the exploration of pregnant women’s perspectives in depth, and in a private environment. The Medical Ethical Committee of VU University Medical Center Amsterdam approved the study protocol.

### Participants and Procedures

Primary care midwives in the Netherlands work independently in privately-owned practices, and are guided by general national and local policies (which do not cover carrier status for HbP). They provide initial prenatal care for 87% of the Dutch pregnant women (Perined 2016). Although HbP carrier screening is not offered routinely in early pregnancy by Dutch midwives (Jans et al. 2012), a few local practices with a high-risk population do offer this test. During February–April 2016, participants were recruited via two midwifery practices (practice A, and practice B) located in an area in Amsterdam with a relatively large population at risk for HbPs, and who are accustomed to offering women HbP carrier screening at the beginning of pregnancy. Participating midwives were informed about the study by a clinical geneticist (PL) and a researcher (KH) before the start of the study. During the booking appointment a number of topics are discussed including medical and obstetric history, lifestyle, family history, and routinely performed blood tests are arranged (e.g. rhesus factor, and possible infectious diseases). Pregnant women were offered additional ancestry-based HbP carrier screening, alongside the standard information on prenatal screening for Down syndrome. At the end of the booking appointment, the midwives provided the women with information about the interview study, and women were asked if they were willing to participate. When participation was declined, reasons for non-participation were asked for. Women willing to participate were introduced to the interviewers by the midwife. Inclusion criteria for women invited into the study were: (1) being at risk for HbPs based on ancestry, i.e. of African, Antillean, Surinam, Asian, Middle Eastern or Mediterranean descent; (2) visiting the midwife for their booking appointment during the current pregnancy; and (3) having been informed by their midwife about HbP carrier screening, and offered the choice to accept or decline screening. Women were not eligible for participation if they were unable to speak either Dutch or English. During the researchers’ presence in the practices, 79 booking consultations were scheduled, of which 42 women were eligible for participation in the interview study, based on ancestry. In total, 26/42 (62%) women were included in the study. The most frequently mentioned reason for non-participation was a lack of time. Six women were counseled by one midwife in practice A, and 20 women were counselled by eight midwives in practice B. Twenty-four interviews were conducted face-to-face, and two by telephone (a few hours, and four days after the intake).

The interview was conducted in a separate room in the midwifery practice directly after the booking appointment. During six of the interviews, the partner was also present but questions were asked to the women. Before the start of the interview, women were provided the opportunity to withdraw themselves from the study at any time, and were explained that there are no right or wrong answers to the questions asked. All participants received a 10-euro gift voucher for their participation. Twelve interviews were conducted by two interviewers (JS and PD; or KH and HH), and fourteen by a single interviewer (HH or KH). The mean duration was 14 min (range 7–25 min). An informed consent form was signed by all participants before the start of the interview, and interviews were audio recorded. Participants’ characteristics are summarized in Table 1.

**Table 1:** Characteristics of participants

	Pregnant women at risk for hemoglobinopathies; *n* = 26
Age (years)	
≤ 25	4
26–35	15
≥ 36	7
Weeks of gestation (range)	10.5 (5–26)
Level of education^a^	
Low/Medium	14
High	6
Unknown	6
Partner (yes)	23
Region/country of origin	
Surinam	13
(South-East) Asia	4
Sub-Saharan Africa	4
Turkey	2
Curacao	1
Afghanistan	1
Uruguay	1
Parity	
Primiparous	12
Multiparous	13
Unknown	1
Previously tested for HbP carrier status (yes), result:	
No carrier	1
Carrier of sickle cell disease	1
Carrier of thalassemia	2
Accepted current HbP carrier screening offer	
Yes	19
No^b^	7

^a^Low: primary school, lower level of secondary school, lower vocational training. Intermediate: higher level of secondary school, intermediate vocational training. High: higher vocational training, university

^b^Two women who already knew their carrier status (1 sickle cell disease, 1 thalassemia) declined the current screening offer for that reason

### Interview Guide

A semi-structured interview guide was developed by the members of the multidisciplinary research team (two health scientists, a clinical geneticist, and a midwife), covering the following topics: familiarity with HbPs and with carrier screening for HbPs; understanding of the meaning of the offer of HbP carrier screening; reasons to accept or decline screening; actions undertaken if found to be a carrier; and opinions about the information provided by the midwife (Online Resource 1).

### Data Analysis

Twenty-five interviews were audio recorded, typed out verbatim, and anonymized; one woman did not give consent for recording, thus anonymized notes were taken instead. A thematic content analysis was performed using the qualitative software program ATLAS.ti version 7.5 for Windows. Coding started by reading all transcripts in detail, and recurring topics were labeled. All labels were clustered into main topics and subtopics in order to identify important themes. In order to increase the credibility of the study negative case analysis was performed (Shenton 2004). In order to safeguard the confirmability of the study, the first four interviews were coded by three researchers independently (KH, HH and LH) and the remaining by two researchers (KH and HH) (researcher triangulation) (Shenton 2004). Differences in coding were discussed until consensus was reached. Representative quotes were translated into English while preserving the character of the original statements, and are used to illustrate the themes. Quotes are accompanied with a description of the participant (participant number, age, gestation in weeks, accepts/declines).

## Results

During the data analysis, four major themes emerged from the interviews: (1) Familiarity with HbPs and carrier screening; (2) HbP carrier screening: reasons to accept or decline testing; (3) a multistep process of decision-making; and (4) perceived information overload during counseling. The findings are summarized below.

### Theme 1: Familiarity with HbPs and Carrier Screening

Four women indicated that they already knew their carrier status (Table 1) before visiting the midwife for their booking appointment, as they had undergone screening after receiving information from their healthcare professional or family members. About half of the participants had previous knowledge, and were familiar with HbPs (mostly SCD) while the others had never heard of it. Of those who had heard of HbPs, most indicated that they had affected family members, friends or acquaintances, while some expressed that they had become familiar with the disease via work, their home country or the media.

*“I’m from Surinam, so I know that Creoles have an increased risk. And I’m a nurse, so I have heard about it before.” (#15, 35 yrs., 7 weeks pregnant, declines)*

Some women who were unfamiliar with HbP explained their unfamiliarity. They argued, for example, that a lack of personal experience with HbP might have influenced the unfamiliarity with both the illness and screening possibilities.

*“I have never heard of it. It doesn’t run in my family, so that’s why it might not come to mind.” (#20, 27 yrs., 9 weeks pregnant, accepts)*

Others explained unfamiliarity in more general terms, explaining that a lack of awareness might be influenced by the rarity of the condition or by a lack of information provided by the healthcare system.

*“So, if there was more attention for it, as for example now with Zika […] but there is little attention for certain diseases, and then people will think, why should I get tested?” (#26, 27 yrs., 8 weeks pregnant, accepts)*

Furthermore, some women said that they had heard of HbP before, but that they did not know about the possibility of carrier screening, and that they never looked into it any further.

*“I didn’t know about it. I did know about sickle cells, but I didn’t know that you could screen for it, or whatever. So I never thought about it”. (#18, 19 yrs., 14 weeks pregnant, accepts)*

### Theme 2: HbP Carrier Screening: Reasons to Accept or Decline Testing

Nineteen women chose to have carrier testing, and seven declined. Although not all women accepted screening, they all generally did perceive the offer of prenatal HbP carrier screening as positive. Women explained that it allowed them to obtain information about the possibilities of screening even if they had not heard about it before, and it enabled people to make decisions.

*“Really good. At least, then you know what risks you’re facing, and you’ll be able to make decisions. So I think it’s good that these tests can be performed.” (#3, 29 yrs., 9 weeks pregnant, declines)*

*“I think it’s good that at least you have the choice if you want to know it. And for myself, I’m kind of a control freak, and yeah, I can imagine that it would be nice if you know it in advance.” (#8, 34 yrs., 8 weeks pregnant, accepts)*

#### Reasons to Accept HbP Carrier Screening

The main reason to accept screening was to obtain knowledge about their own carrier status, and about the health of their unborn child.

*“If I have that knowledge, it’s only good, right? Probably it’s not the case [being a carrier], the chances are really small, at least that is what the midwife told us. But when you have that knowledge, you can make a better decision, I think.” (#21, 32 yrs., 9 weeks pregnant, accepts)*

The ease of the procedure was also often mentioned as a reason to accept HbP carrier screening. Women argued that screening is performed simultaneously with other blood tests, and it thus entails only one extra sample of blood.

*“Honestly, I thought: well, I’m going to have my blood drawn anyway, so one extra sample, why not?” (#10, 28 yrs., 8 weeks pregnant, accepts)*

During the interview, one woman argued that she did not want to be screened, as this does not provide any certainty, and because she felt that nothing could be done when her unborn child turned out to be affected. However, when completing blood examination forms, she did choose to have testing in the end, as she was having other tests as well.

*“Actually, it doesn’t say anything, so basically, what do you really know in the end? So in that case I don’t want to test. But why shouldn’t I do it as I’m already having my blood drawn for other tests anyway?” (#11, 39 yrs., 9 weeks pregnant, accepts)*

Another woman explained that her previous experiences with a miscarriage influenced her decision to accept the screening offer. Where in the past she had declined different tests, she would like to be screened now.

*“This isn’t our first pregnancy, it’s our second. During the first we said: well we’re not going to do that […] we were not going to have the first trimester combined test, we were also not going to have HbP carrier screening, and we were like, it’s our first pregnancy, let’s go for it. But then it went wrong, and now we look at it a little bit different”. (#10, 28 yrs., 8 weeks pregnant, accepts)*

One woman remarked that she chose to accept testing because she was already a bit older but also because she accepted other tests as well. HbP carrier screening then only entails one extra test.

*“[…] Yeah, because I’m already 44 years old, and if you want it anyway, you might test as much as possible. Look, there is the first trimester combined test, and then you might think, let’s have everything now. Then you’ll know how things are.” (#23, 44 yrs., 10 weeks pregnant, accepts)*

For some women, the importance of testing for their own health was also a reason to have carrier screening. They acknowledged that it would provide knowledge about their own health, and that sometimes being a carrier of HbP could have consequences for them as well.

*“[…] Eh, because with all my previous pregnancies I suffered from an iron deficiency and anemia, so yeah, I really would like to know what it is.” (#22, 30 yrs., 12 weeks pregnant, accepts)*

Finally, one woman remarked that the she has been guided by her midwife in deciding to accept screening or not.

*“For me I’m not a professional on how the pregnancy will go, so I trust my midwife or my general practitioner to do anything they tell me.” (#19, 33 yrs., 10 weeks pregnant, accepts)*

#### Reasons to Decline HbP Carrier Screening

Few women declined HbP carrier screening. One women argued that there was no need for HbP carrier screening as HbPs did not run in her family and that she already has healthy children.

*“Well, you know, I already have two healthy children and it doesn’t run in my family. And I don’t think my boyfriend has it.” (#15, 35 yrs., 7 weeks pregnant, declines)*

This particular woman furthermore mentioned that, though being aware that she could not know her carrier status for certain without testing, she declined screening because her feeling told her that she was not a carrier.

*“Well, I just have the feeling that I’m not a carrier. I obviously know that you can’t know it for sure, but I just think that it’s not the case with us.” (#15, 35 yrs., 7 weeks pregnant, declines)*

Another woman declined screening because of her fear of needles, and she mentioned that other tests during the pregnancy (ultrasound) also provide knowledge about their unborn child. As she had understood from the midwife that the neonatal screening also provides information about HbP, she preferred to wait for those results. Furthermore, some women indicated that they (or their partners) had already been tested in the past, and declined screening for that reason.

*“He’s been a blood donor, and there they already ruled out that he was a carrier”. (#16, 31 yrs., 7 weeks pregnant, declines)*

Other reasons expressed to decline screening were the fact that carrier screening would not provide certainty about the condition of their unborn child, or that their partner is not at increased risk of being a carrier.

### Theme 3: A Multistep Process of Decision-Making

One of the key findings from the interviews was that for many women decision-making seemed to be a multistep process. They indicated that they did not want to think about the possible consequences of a positive (unfavorable) test result when they made their initial decision on accepting or declining carrier screening. Women argued that they would like to wait for the results of their carrier screening test before thinking about and deciding on follow-up testing.

*“At this moment, I’ll just see what happens, and when we do this test, and it turns out that I’m a carrier, then I would like to proceed with other tests of course. In the end, it’s all about my little one.” (#10, 28 yrs., 8 weeks pregnant, accepts)*

*“I’m like, I’ll see what happens and I’ll take it from there if I turn out to be a carrier.” (#20, 27 yrs., 9 weeks pregnant, accepts)*

Despite the multistep process of decision-making illustrated above, one woman did acknowledge that the need for follow-up testing of their partner (or invasive diagnostic testing) crossed her mind while deciding for or against screening. She argued that this was something to bear in mind.

*“You’ll have to remember that you will not know it for sure because you should examine it further.*” *(#11, 39 yrs., 9 weeks pregnant, accepts)*

#### Testing of the Partner

Even though women did not give follow-up testing much consideration while deciding to participate in carrier screening, about half of the test acceptors indicated during the interview that they would like to have their partner tested as well. Reasons to have their partner tested were comparable with their own choice for screening. They indicated that the testing of their partner was important to obtain more knowledge about the consequences for their unborn child, as one woman mentioned:

*“Well, then you know whether he’s also a carrier or not. That’s just important. Then you’ll know, OK, he’s a carrier, I’m a carrier, and then you’ll know that your child… it’s just important!” (#26, 27 yrs., 8 weeks pregnant, accepts)*

Another woman mentioned that her husband had an autoimmune disease, and she expected that, because of the experience with having a disease, he would like to have HbP carrier screening as well.

*“Well, we discussed it yesterday by chance. My husband has this skin condition […] he’s suffering from this kind of autoimmune thing. […] So I guess he would like to know this [carrier status] as well, to be sure.” (#8, 34 yrs., 8 weeks pregnant, accepts)*

Some women were undecided about having their partner tested, while others declined partner testing for several reasons. In some cases, women explained that their partners had already been tested because previous carrier testing indicated that she was a carrier. Furthermore, a few women stated that their partners were staying abroad or that the relationship had already ended. In light of this latter comment, a participant expressed the wish to be able to test without needing to test the father as well.

*“But what if it [risk for her unborn child] could already be examined without him, I don’t know how, but I would choose to do that. As long as it’s not too complicated.” (#5, 36 yrs., 15 weeks pregnant, accepts)*

#### Attitudes towards Invasive Diagnostic Follow-Up Testing and Termination of Pregnancy

When a carrier couple is identified during pregnancy, they can opt for invasive diagnostic testing (amniocentesis or a chorionic villus sampling (CVS). In line with the multistep process of decision-making, women seemed not to give these procedures much consideration. However, important factors that seemed to shape women’s attitudes towards invasive diagnostics, and which might be considered when making a decision, were identified.

The first factor was related to the conditions tested for. Women frequently mentioned the perceived severity of the condition of being of importance in decision-making for invasive diagnostics or termination of pregnancy (TOP). Some women thought that HbPs were not severe enough to consider both invasive diagnostics and TOP. Although uncertain about the quality of life of affected children, one woman would base her decision for subsequent procedures on her assumption that quality of life was not as severely affected as with other diseases.

*“The chorionic villus sampling is, of course, not only performed for sickle cell disease but also for Down syndrome or other abnormalities. I would not consider having a chorionic villus sampling for sickle cell because I think, and that’s something I feel but I’m not sure about, life with it [SCD] is somewhat easier than with some other kind of disability.” (#16, 31 yrs., 7 weeks pregnant, declines)*

Another woman also argued that the quality of life, and the impact of the disorder, is relevant when deciding on having invasive diagnostics and TOP.

*“Look, I would only have an amniocentesis if I was going to consider an abortion. And I would only consider an abortion if the impact of the disorder was so huge that it would be difficult to take care of.” (#19, 33 yrs., 10 weeks pregnant, accepts)*

While some would like to perform invasive prenatal testing to be able to prepare for the birth of an affected child, others felt that nothing could be done during pregnancy in terms of treatment. One woman explained that having invasive diagnostics would only induce anxiety and stress, which would have a negative impact on her pregnancy. Furthermore another woman added that the child could also be tested for HbPs after being born.

*“As understood from my midwife, neonatal screening will also provide information about the baby.” (#14, 21 yrs., 26 weeks pregnant, declines)*

Test safety was found to be of importance in shaping women’s decision-making. Women felt that the miscarriage risk when having invasive diagnostics was unacceptable and some refused to have testing for that reason. Others formulated this differently, by stating that invasive diagnostics might intrude in the development of the fetus.

*“Well he’s still growing and developing. As far as I’m concerned nothing goes inside my belly. It might trigger other things to happen… it will disturb the natural rhythm of developing.” (#26, 27 yrs., 8 weeks pregnant, accepts)*

One woman felt that the time between invasive diagnostics, and the possible decision for TOP, was too long to be able to make this decision.

*“But if you are already that far along, and you will then have to have all these tests, it will take a couple of weeks. Lots of things will happen with your body during that period, and I think, for me personally, I think that it is too late then.” (#11, 39 yrs., 9 weeks pregnant, accepts).*

Finally, moral or religious beliefs around TOP were also frequently mentioned by participants when reflecting on invasive diagnostics. Some women felt that a child is given by God, and that it is the parents’ responsibility to take care of the child, and they would decline invasive testing and TOP for that reason. Another woman argued that she had waited so long for this pregnancy that she felt that any child was welcome, no matter what.

*“Well, we’ve been waiting all this time for a little one. You know, I’m 34, almost 35 years old, and at this moment I can’t imagine that it would be so horrendous that I wouldn’t want this baby.” (#8, 34 yrs., 8 weeks pregnant, accepts)*

### Theme 4: Perceived Information Overload during Counseling

In general, women were positive about the information provision and counseling about HbP carrier screening provided by their midwife. However, they also felt that some things could be improved, such as the information provision and its timing. Topics needing more explanation entailed, for example, the consequences of HbP for the health of their unborn child, the reasons for being at an increased risk, and more information about why certain ethnic groups are offered screening.

*“Well the reason why this is tested among different ethnic groups. […] But again, I would like know what the consequences are for my child, yeah.” (#9, 37 yrs., 9 weeks pregnant, accepts)*

In contrast, other women perceived an information overload during the booking appointment, and felt that there was not enough time to receive sufficient information about HbP (carrier screening). Some explicitly felt that this particular appointment is not the designated moment to embed counseling and information about HbP (carrier screening) as many topics are already discussed here. As a result of this, information might not be adequately absorbed by women or might cause a feeling of being overwhelmed by all the information together.

*“You’ll get so much information during this booking appointment, it just went a little bit past me.” (10, 28 yrs., 8 weeks pregnant, accepts)*

*“On the one hand, I was struck by it [information about HbP carrier screening]. Because I thought, that’s another test I have to do, with additional costs involved. At first I thought about what we would do if there was an increased risk of having a child with Down syndrome, and this is something new. I thought it was [sighs] a bit much at that moment.” (#9, 37 yrs., 9 weeks pregnant, accepts)*

Women suggested that it would be better to have a separate counseling moment for this purpose such as an information evening. They furthermore argued that information about HbP and carrier screening could be provided by midwives or general practitioners, and could also be spread using leaflets.

*“Actually, I would have liked to know it in advance because it seems like it’s not really common. […] Maybe the midwife can introduce it on their website, or in some paperwork we can read before we go to the booking appointment. […] Maybe they can send us some information in advance? (#19, 33 yrs., 10 weeks pregnant, accepts)*

Some women felt that the timing of the offer (prenatal) was not appropriate. It was expressed that there were so many things to think about during a pregnancy, and it would therefore be preferable to test before the pregnancy (preconception).

*“I think maybe, if that’s possible, that you would want to test earlier, before you’re pregnant. Because, now you’re overwhelmed by all these tests, and that’s not what you want because you already have so much to think about. So when you have the opportunity to test early, for example when you and your partner are planning to have children, it would be good to do it then.” (#10, 28 yrs., 8 weeks pregnant, accepts)*

Other options mentioned were neonatal screening, or offering screening during childhood.

*“Well, considering that some people find out about it [being a carrier] during their pregnancy, it would be better to test at a different moment. But when? Maybe if the children are still young, they get all those injections and tests, right? Maybe they should test it then.” (#5, 36 yrs., 15 weeks pregnant, accepts)*

## Discussion

This study showed that most pregnant HbP high-risk women (i.e. women considered at higher risk of being a HbP carrier based on their ancestry) perceived the offer of HbP carrier screening by their midwife as positive, and accepted the screening offer. Important reasons to accept screening were to obtain knowledge about their own carrier status and indirectly about the health of their unborn child, and the ease of the procedure. Reasons to decline screening included: the absence of a positive family history, having the feeling of not being a carrier, and a fear of needles. A multistep process of decision-making was observed, as many women did not give follow-up testing of their partner and/or prenatal testing much consideration in the case of screen-positive results while deciding on accepting or declining HbP carrier screening. Though a need for more information was expressed by some women, others experienced an information overload as the information came unexpectedly. Women preferred receiving the information and the screening offer at a different moment (e.g. before the intake by means of a leaflet or at an information evening, or even preconceptionally).

This study showed that half of the respondents were familiar with HbP and/or HbP carrier screening as has also been shown by van der Pal et al. (2013). However, it has also been shown that few people know HbP to be endemic in their home country, which might partly explain women’s unfamiliarity with HbPs (van der Pal et al. 2013).

The positive attitudes toward HbP carrier screening in general, and during early pregnancy specifically, seen in our participants, are in accordance with other studies in, for example the UK (Ahmed et al. 2002; Brown et al. 2011; Tsianakas et al. 2012) and in the Netherlands (van der Pal et al. 2013). The willingness to be screened also shows parallels with the literature (Brown et al. 2011; Giordano et al. 2006; Housten et al. 2016; Lakeman et al. 2009; van der Pal et al. 2013; van Elderen et al. 2010). Although studies that have been published on specific reasons to accept or decline HbP carrier screening are sparse, the perceived relevance for future children and obtaining information or reassurance were reasons to accept screening described in the literature (Lakeman 2008; van der Pal et al. 2013). Feeling healthy and the absence of a positive family history were reasons to decline HbP carrier screening (Lakeman 2008; van der Pal et al. 2013). Furthermore, reasons to accept or decline also show similarities with literature on carrier screening for other disorders, such as CF. In their review on CF carrier screening, Ioannou et al. (2014) described various reasons to accept or decline screening, including reasons comparable with our findings. Accepting carrier screening due to the ease of the procedure is one argument to pay specific attention to. Though it is positive that HbP carrier status can be determined relatively easily, it is important that women think carefully about the offer of carrier screening and its consequences. Decisions should not solely be guided by the ease of the procedure to avoid the possible routinization of screening. In the literature on the evaluation of high-school carrier screening in the Ashkenazi Jewish population, a fear of needles was found to be a major concern, with a significant proportion of students declining screening for that reason (Gason et al. 2003). However, in another study on HbP carrier screening, this was of no importance (Lakeman et al. 2009), and in our study, only one woman declined screening for this reason.

The multistep process of decision-making observed in our study has also been described in the literature on prenatal screening for Down syndrome, although explained differently. In the study by Lewis et al. (2016), pregnant women were provided with information about the possibility of non-invasive prenatal testing (NIPT) at multiple moments in time (e.g. at the booking appointment, after receiving the Down syndrome screening results). Two types of women could be identified. Those who already made an initial judgement about the relevance of NIPT but were not yet required to make a decision at their booking appointment, which was helpful for deliberation. And those who did not think ahead and considered NIPT only when it became relevant to them (Lewis et al. 2016). This second type shows parallels with our study, as women primarily considered having HbP carrier screening while postponing deliberating or deciding on possible follow-up tests. It has been shown that not thinking about possible consequences and follow-up tests when making an initial decision might lead to suboptimal informed decision-making (Crombag et al. 2016). Efforts to improve this, for example by providing information at multiple moments, would therefore be important.

Though not explicitly measured, it seemed that women’s knowledge was not optimal which might jeopardize informed decision-making. As described by Marteau et al. (2001), an informed decision is “a decision made with sufficient knowledge, consistent with the decision-maker’s values and behaviorally implemented”. However, there are no agreed thresholds for the knowledge an individual is required to have to make an informed decision, thus hampering its measurement (Ames et al. 2015). Some women expressed not understanding all information provided by the midwife, and some provided incorrect information during the interview. To illustrate this, one woman argued that she accepted screening partly due to her advanced maternal age, whereas another woman declined screening because of the absence of a positive family history for HbPs. Advanced maternal age is, however, not a risk factor for being a HbP carrier, and in the majority of the cases, autosomal recessive disorders occur in families where no children with that specific disorder have been born before (Henneman et al. 2016). This observation corresponds with the findings of Brown et al. (2009) in the UK where two-thirds of the pregnant women made an uninformed choice about HbP screening due to poor or incorrect knowledge.

Evaluating the offer, the question arises whether adequate conditions for informed decision-making regarding carrier screening were present, possibly resulting in the described multistep process of decision-making. Carrier screening was offered during the booking appointment, in which multiple topics are discussed, varying from lifestyle issues to counseling for the first trimester combined test to screen for Down syndrome. Since information provided on HbP carrier screening only entailed a small part of this appointment, women perceived information overload during this appointment. By contrast, they also expressed a need for more information on the topic of HbP. This need has also been described by others (Cousens et al. 2013; Tsianakas et al. 2012), and this lack of information and suboptimal knowledge might influence the informed decision-making process and consent. In Australia, carrier screening for thalassemia is one of the objectives of routine prenatal tests, and carriers are identified as a result of a full blood examination (Cousens et al. 2014; The Royal Women’s Hospital 2015). Strikingly, women do not appear to be providing informed consent for this particular form of genetic screening, and are often surprised by the test results (Cousens et al. 2013). In the absence of a structural offer of HbP carrier screening together with structural guidance and education for health professionals in the Netherlands, some professionals also suggested integrating HbP carrier screening in existing routinely performed blood tests during pregnancy (Holtkamp et al. 2017). It is however important to bear in mind that the aim of these routine blood tests differs from that of carrier screening: monitoring pregnancy versus facilitating reproductive choice. Combining these two tests, and thus combining two different aims of testing, can be ethically challenging, as has also been described in the context of prenatal screening (Dondorp et al. 2016). Sufficient information provision and facilitating informed decision-making and consent should be guaranteed.

Reflecting on the reproductive options available, especially invasive diagnostics and termination of pregnancy (TOP) in the case of an unfavorable result of the carrier screening test, only a few women indicated that they would consider those options. Others argued that they would refrain from this, for example because they felt nothing could be done during their pregnancy, or because a child is a gift from God. Research on a hypothetical screening offer in the Netherlands showed that one-third of the study participants would opt for prenatal diagnosis if screening confirmed carrier status for HbPs for both partners, and of this group, one-third would consider TOP in the case of an affected pregnancy (van Elderen et al. 2010). Lakeman et al. (2008) furthermore showed that about half of their screening study participants would consider TOP. Though data on TOP are unpublished, experiences with prenatal HbP carrier screening in the UK showed that about 40% of *n* = 944 identified carrier couples opted for prenatal diagnosis (National Health Services England 2015). These numbers show that some of the women do intend to act upon the carrier screening test results, either for preparatory purposes or to guide decisions on invasive diagnostics and TOP. In the present study, women furthermore indicated that screening could also be performed at different moments in time, e.g., preconceptionally or during childhood. As part of current neonatal screening, affected children are diagnosed, and carrier status for HbS found incidentally is also reported. More research is needed on the perspectives of high-risk women and the population at risk, and their needs in terms of information and screening in several phases of life.

### Study Limitations and Research Recommendations

One of the strengths of this study is its qualitative approach. By conducting semi-structured interviews, it was possible to study women’s perspectives on prenatal HbP carrier screening in depth. Furthermore, we were able to engage a target group that is often difficult to reach for scientific research purposes. This might be due to the research method chosen, the incentive for participation, or the timing of the interviews. Interviews were conducted directly after the booking appointment to minimize the burden and time constraints for women. However, the timing might also be a limitation of this study. Some women had other appointments directly after their booking appointment, and declined participation for that reason. Telephone interviews might be a solution here. Another limitation is that the interviews were relatively short. This could also be due to the timing of the interview, but it was also observed that there was a need for closed-ended questions among women, which might have resulted in less extensive conversations. Additionally, the interviews showed that women’s knowledge was not always optimal, and a need for more information was expressed. Unfortunately, it was not possible to study whether counseling by the midwives played a role here. An observational study in which a number of booking appointments are attended could provide more insight into this process. Moreover, due to practical considerations (researchers available and their language skills) only women who could speak Dutch or English could be included in the study which might have resulted in the loss of valuable information of first-generation immigrants at risk of being a HbP carrier. However, of those women non-eligible for participation, in only three cases (out of 37 non-eligible women) exclusion was based on the language barriers. Finally, this qualitative data should not be used for generalization, and future studies should therefore be quantitative to confirm our findings.

### Conclusion and Practice Implications

The findings from this study provide insight into how HbP high-risk pregnant women perceive an offer of prenatal HbP carrier screening by their midwife. Though not all women chose to accept screening, the offer was perceived as positive. A variety of reasons to accept or decline carrier screening was identified. Furthermore, decision-making seemed to be a multistep process, as many women did not give follow-up testing much consideration early on in the process. Additionally, informed decision-making seemed to be suboptimal. More attention should be given to the key functions of counselling: health education and decision-making support (Martin et al. 2013). In light of this, women indicated that the content as well as the timing of both the information provided and the actual offer needs improvement, which can, for example, be realized by providing information (in writing) at different moments in time prior to the booking appointment or even preconceptionally.

## Electronic supplementary material

ESM 1 (PDF 266 kb)
