# Supplementary material for: Experiences of a High-Risk Population with Prenatal Hemoglobinopathy Carrier Screening in a Primary Care Setting: a Qualitative Study
Source: J Genet Couns. 2017 Oct 5;27(3):635–46. doi: 10.1007/s10897-017-0159-7 (PMC5943376; doi:10.1007/s10897-017-0159-7)
Supplement: Supplementary file 1 — (PDF 266 kb) [file 10897_2017_159_MOESM1_ESM.pdf]

Article title: *Experiences of a high-risk population with prenatal hemoglobinopathy carrier screening in a primary care setting: A qualitative study*

Journal: Journal of Genetic Counseling

Authors: Kim C.A. Holtkamp[1], Phillis Lakeman[2], Hind Hader[1], Suze M.J.P. Jans[1,3], Maria Hoenderdos[4], Henna. A.M. Playfair[5], Martina C. Cornel[1], Marjolein Peters[6], Lidewij Henneman[1]

[1]Department of Clinical Genetics, section Community Genetics, Amsterdam Public Health research institute, VU University Medical Center, Amsterdam, The Netherlands
[2]Department of Clinical Genetics, Academic Medical Center, Amsterdam, The Netherlands
[3]TNO, Quality of Life, Department of Child Health, The Netherlands
[4]Midwifery practice Vida, Amsterdam, The Netherlands
[5]Midwifery practice Bijlmermeer, Amsterdam, The Netherlands
[6]Department of Pediatric Hematology, Emma's Children's Hospital, Academic Medical Center, University of Amsterdam, The Netherlands

Corresponding author e-mail: l.henneman@vumc.nl

Online Resource 1. Semi-structured interview guide

[Introduction]

Good morning/afternoon, I am [*name*]. You have just had your booking appointment with the midwife, could I ask you a few questions regarding this in the context of a study?

[*If yes, thank you very much for your cooperation. We could take a seat over here to talk in private. If not, could you maybe briefly indicate why you don't want to participate?*]

[*Note.* *When a pregnant woman brings her partner, indicate that you have some questions for her. Ask the partner if he/she wants to join as well. If yes, clearly state this in the documentation.*]

May I congratulate you with your pregnancy? Is it your first pregnancy? How are you feeling? Thank you for participating in this conversation, it will take approximately 20 minutes. I will first explain why I would like to speak with you.

We are collaborating with the midwife, and the conversations we have aim to identify what people know about sickle cell, and testing for this purpose. I am curious to know whether you have heard about this, and what you know about it.

- I am looking for your opinion; there are no right or wrong answers.
- Everything you tell me during this conversation will be treated confidentially, and will not be discussed with your midwife. This conversation will be processed into a report; your name and other personal details will not be included.
- With your permission, this conversation will be audio recorded in order to process and analyze it properly. The records will be destroyed after processing.
- At the end of the conversation, you will receive a gift voucher to thank you for your time.

Do you have any questions at the moment? Then I would like to ask you to sign this informed consent form.

[*Signing of the informed consent form*]

I would like to start with the questions now.

[*Start audio recording*]

[Questions]

Information about hemoglobinopathies (HbP) during counseling with midwife.

1. Did your midwife just explain something to you about sickle cell and/or thalassemia and being a carrier?
    a. *If yes, what did your midwife tell you? Can you tell me something about that?*
    b. *Do you know what it means when you and your partner are both carriers of sickle cell? Can you tell me something about that? What do you know about that?*
    c. *Are you satisfied with the information the midwife gave you? Why, why not?*
    d. *What other or additional information would you have liked to receive? What other questions do you have?*

Familiarity with HbP

2. Had you ever heard about sickle cell disease or thalassemia before?
    a. *If yes: what do you know about this?*

3. Did you know that people from Africa, Surinam, Antilles, and the Caribbean have an increased risk of being a carrier of sickle cell?

4. Do you know someone with sickle cell?
    a. If yes, who (family/friends)?

5. To what extent has this topic been discussed in your family, or with others? Why/why not?

6. In your opinion, to what extent is it possible to have a good life with hemoglobinopathies? Why/why not?
    a. *Do you know how a child gets an HbP? Can you explain that?*
    b. *Do you think you have a chance of being a carrier of an HbP or of having a child with an HbP? Why/why not?*

Carrier screening

7. To find out whether there is a chance of having a child with an HbP, there are tests available. Did your midwife tell you something about this? Or was this test offered to you?
    a. Are you having a test to find out you are a carrier? Why/why not?
    b. What are you going to do with the test results?

8. What have you been told that you can do when the test shows that you are a carrier of an HbP?
    a. *To what extent did your midwife tell you something about the possibility to test the baby's father? Can you tell me something about that?*
    b. *Do you think that you would have the baby's father tested? Why/why not?*
    c. *Do you think the baby's father wants to get tested? Why/why not?*

9. Suppose you and the baby's father are both carriers of sickle cell.
    a. What would you do with the results?
    b. In what way do you think this might affect your baby?
    c. Do you know how you can find out whether your baby has an HbP?
    d. Would you consider this test to examine the baby? Why/why not?
    e. What is your opinion about termination of pregnancy (abortion) in the case of HbPs?
    f. Would you consider this yourself if you find out your baby has an HbP? Why/why not?

10. What do you think about having this test during your pregnancy?

[Closure]

To conclude, I have a few more questions.

- How old are you?
- How many weeks are you pregnant?
- Is this your first pregnancy? If not, how many children do you have?
- Where were you born? Where were your parents born? And your partner?

- Is there anything I forgot to ask that you think should be discussed?
- Do you have any questions?

I would like to thank you very much for this conversation!

[*Turn audio recorder off, and hand over the gift voucher*]
